# Allelic loss at 19q12 and Xq11–12 predict an adverse clinical outcome in patients with mucinous ovarian tumours of low malignant potential

**DOI:** 10.1038/sj.bjc.6601681

**Published:** 2004-03-02

**Authors:** K Nakayama, Y Takebayashi, K Hata, R Fujiwaki, K Iida, M Fukumoto, K Miyazaki

**Affiliations:** 1 Department of Obstetrics and Gynecology, Shimane Medical University, Enyacho 89-1, Izumo 693-8501, Japan; 2 Department of Surgery II, Institute of Biomedical Sciences Fukushima Medical, University School of Medicine, Japan; 3 Department of Pathology Institute of Development, Aging and Cancer, Tohoku University, Sendai, Japan

**Keywords:** ovarian carcinoma, mucinous LMP, loss of heterozygosity, prognosis

## Abstract

Ovarian tumours of low malignant potential (LMP) are intermediate between adenomas and ovarian carcinomas. These tumours are often associated with a significantly better prognosis than ovarian carcinomas. However, a subset of these tumours can progress and become lethal. In order to seek sensitive diagnostic tools for monitoring patients after surgical operation, we performed a genome-wide scan for loss of heterozygosity (LOH) in 41 mucinous LMPs using 91 polymorphic microsatellite markers at an average interval of 50 cM across all of the human chromosomes and 25 LOH markers reportedly associated with ovarian carcinoma. In addition, we assessed whether clinicopathological parameters, microvessel density, Ki-67 labeling index, apoptotic index or p53 overexpression would be useful for predicting the postoperative outcome of LMP patients. Of the 116 markers examined, 19q12 and Xq11–12 showed significant correlation between postoperative progression-free survival time and LOH status (*P*<0.05). Patients with a high Ki-67 labeling index had a significantly poorer progression-free survival time than those with lower levels (*P*=0.042). Other clinicopathological factors and immunohistochemical analysis had no correlation with progression-free survival time in this series of patients. When the combination of LOH at 19q12 and/or Xq11–12 was assessed using Cox's regression analysis, patients with tumours that showed LOH at these positions were at greatest risk of progression (*P*=0.0073). These findings suggest that the identification of LOH at 19q12 and/or Xq11–12 in former mucinous LMP sites should alert the clinician to the presence of a potentially aggressive lesion in the coelomic epithelium, even if a distinction between second primary tumours or recurrence could not be determined.

Ovarian carcinoma is the most lethal gynaecological malignancy at present (Wingo *et al*, 1995). In 1929, Taylor first recognised the existence of a group of ovarian carcinomas that were associated with an improved prognosis; he termed these lesions ‘semimalignant’ tumours (Taylor, 1929). In 1961, the International Federation of Gynaecology and Obstetrics (FIGO) proposed a classification of ovarian tumours that included low malignant potential (LMP) lesions; this designation became effective in 1971 (FIGO, 1987) and was incorporated into the World Health Organisation classification in 1973 (Serov *et al*, 1973). Characterised by complex branching papillae, epithelial stratification, nuclear atypia, mitotic activity, and absence of stromal invasion, these tumours are considered to be of LMP. Serous and mucinous tumours comprise the vast majority of cases, with endometrioid, clear cell, and Brenner-type tumours also described (Russel *et al*, 1994). Low malignant potentials (30–50%) are mucinous type (Lee and Scully, 2000) and the incidence of mucinous LMPs is high in Japan compared to Western countries (Nakashima *et al*, 1990; Tamakoshi *et al*, 1997). Mucinous LMPs present at earlier stages than invasive ovarian carcinomas and have a more indolent long-term course and excellent prognosis after surgical management alone (Tazelaar *et al*, 1985; Casey *et al*, 1993; Trimble and Trimble, 1994; Siriaunkgul *et al*, 1995; Gershenson, 1999; Lee and Scully, 2000). Mucinous LMP patients (60–85%) who present with stage I disease can be managed conservatively with unilateral salpingo-oophorectomy and complete staging. The postoperation 10-year survival rate is 93–99% for stage I disease (Casey *et al*, 1993; Trimble and Trimble, 1994; Gershenson, 1999; Lee and Scully, 2000). For the 15–30% of patients who present with stage II–III disease, TAH-BSO and surgical staging are the treatments of choice. Approximately 10–30% of these patients will relapse and 10% will die of the tumour. The criteria for adjuvant chemotherapy for LMP patients are still undetermined. An estimated 10% of patients will develop ovarian carcinoma (Lee and Scully, 2000). Whether these invasive secondary tumours represent progression of LMP to invasive disease or new secondary tumours arising in at-risk populations is still controversial (Siriaunkgul *et al*, 1995; Lee and Scully, 2000). However, a subset of these tumours can progress and become lethal. This poor outcome is largely due to a lack of sensitive diagnostic tools for monitoring patients after surgical operation, with a reliance upon clinical and pathological parameters that are often difficult to assess for therapeutic decision making. In the search for more reliable prognostic indicators, a few investigators have focused on biological markers that might be predictive of LMP development (Kaern *et al*, 1990; Padberg *et al*, 1991). For example, Kaern *et al* (1990) reported that DNA ploidy pattern was the most important prognostic marker in their study. However, until now it has not been known which molecular markers influence the fate of these patients, because little is known about genetic alterations in LMPs. Among the various types of genetic alterations involved in ovarian carcinoma development and progression, loss of heterozygosity (LOH) of particular chromosomal regions is thought to indicate the deletion of a normally resident tumour suppressor gene (Callahan and Campbell, 1989; Knudson, 1993). Thus, it is possible that the loss of specific alleles may act as diagnostic markers of prognosis. Recently, we reported that allelic loss could be a predictor of poor outcome for ovarian carcinoma patients (Nakayama *et al*, 2003b). In order to seek potential predictive markers in the current study, we performed a genome-wide scan for LOH in 41 mucinous LMPs using 91 polymorphic microsatellite markers at an average interval of 50 cM throughout all of the human chromosomes (screening markers) and 25 LOH markers reportedly associated with ovarian carcinoma (hotspot markers). In addition, we assessed whether various clinicopathological parameters, microvessel density, Ki-67 labeling index, apoptotic index (AI), or p53 overexpression would be useful for predicting the postoperative outcome of mucinous LMP patients.

## MATERIAL AND METHODS

### Patients and tumour samples

In all, 41 mucinous LMPs and their adjacent non-neoplastic tissues were obtained from archival pathological specimens at the Institute of Japan Surgical Pathology and Seirei Hamatsu General Hospital in Japan. Written informed consent for the analysis in this study was obtained for each case individually. Diagnostic verification and tumour subtyping and grading were performed independently by three certified pathologists (TN, NT, and MF). Low malignant potentials were diagnosed on the basis of conventional histopathologic criteria (Serov *et al*, 1973), and grading criteria recommended by the International Federation of Gynecology and Obstetrics were used.

The ovarian origin of advanced-stage tumours was confirmed by endoscopic examination, low levels of carcinoembryonic antigen, and the clinical course, as well as by consultation with gastrointestinal specialists of internal medicine and surgery, and urologists. The present tumour tissues consisted of 41 mucinous LMPs. There were 30 stage Ia patients, one stage Ib patients, eight stage Ic patients, two stage II patients, and one stage III patient in this study. Clinicopathological characteristics of these cases are summarised in Table 1

**Table 1:** Summary of clinical stage and histological subtype of LMPs

	**Stage**^a^					
**Histology**	**Ia**	**Ib**	**Ic**	**IIa**	**IIb**	**IIIc**
Mucinous	30	1	7	1	1	1

a Clinical staging was determined according to the International Federation of Gynaecology and Obstetrics.

LMP=low malignant potential.

.

Unilateral salpingo-oophorectomy and TAH-BSO were performed on patients who presented with stage I disease and with stage II–III disease, respectively. None of the patients received adjuvant chemotherapy. Follow-ups for all patients included in the survival analysis were updated in January 25, 2003 (median follow-up time was 103 months; range, 60–144 months). At that time, six patients had progressed to well-differentiated ovarian carcinoma, one patient had progressed to *Pseudomyxoma peritonei*, and 34 had not progressed.

### DNA extraction

**Figure 1:**
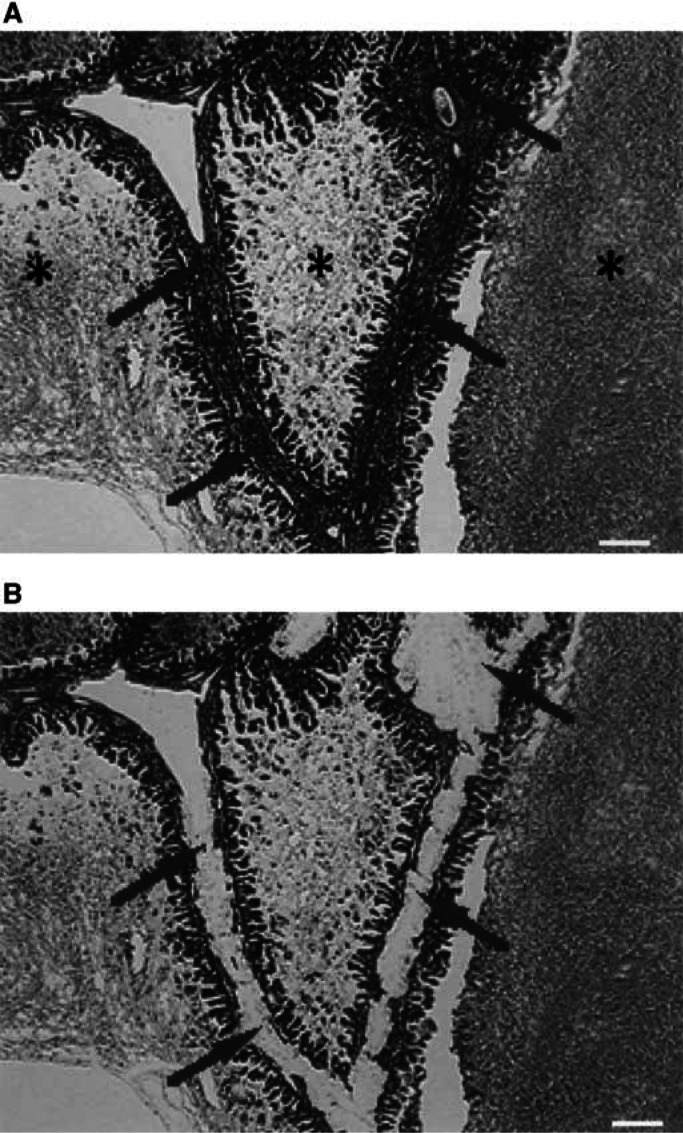
(**A**) An LMP tumour before microdissection (× 20, H&E). Arrows indicate stromal cells and connective tissue. ^*^, mucinous substrate without cellular component. Scale bar, 25 *μ*m. (**B**) The LMP portion after the removal of normal components. The LMP was collected for DNA extraction (× 20, H&E). Arrows indicate removed stromal cells and connective tissue. Scale bar, 25 *μ*m.

**Figure 2:**
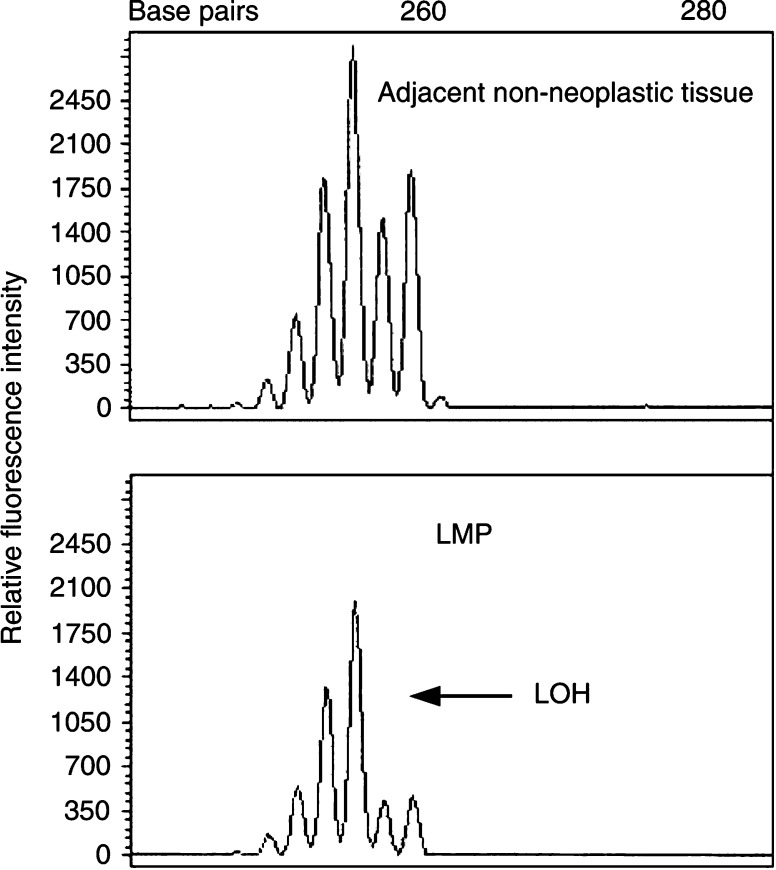
Representative example of LOH.

Paraffin-embedded tissues were sectioned at a thickness of 5 *μ*m and stained with haematoxylin and eosin. The cancerous and non-neoplastic portions were collected separately with a 29-gauge needle using an MK1 micromanipulator (Singer Instruments, Roadwater, UK) under a dissecting microscope (Figure 1

). The dissected tissue was collected in an Eppendorf tube and incubated overnight at 58°C in a digestion mixture (0.01 M NaCl; 0.5 M Tris-HCl, pH 8.0; 20 mM EDTA; 0.05% Tween-20R; 0.1 mg ml^−1^ proteinase K). The samples were then heated to 95°C for 10 min to inactivate the proteinase K activity. After the digestion, DNA was extracted with phenol/chloroform treatment and ethanol precipitation. A representative example of LOH is shown in Figure 2

.

### Microsatellite markers

We analysed LOH in LMPs with dye-labelled microsatellite markers, including 25 markers for which LOH is reportedly frequent in ovarian carcinoma (hotspot markers). Primer information for the hotspot markers has been described previously (Nakayama *et al*, 2001). Further LOH analysis in LMPs was carried out with 91 markers located at an average distance of 50 cM apart and distributed throughout all of the human genome (screening markers) (CHLC/Weber Human Screening Set 6A, Research Genetics, Huntsville, AL, USA; primer information can be obtained from K Nakayama).

### PCR amplification and analysis of LOH

PCR reactions were performed in a total volume of 10 *μ*l containing 25–50 ng of DNA, dNTPs at a final concentration of 20 *μ*M, 0.4 *μ*M of each primer, and 0.25 U of *Ex-Taq* DNA polymerase (Takara Shuzo, Shiga, Japan) or Platinum *Taq* DNA polymerase (GIBCO BRL, Rockville, MD, USA). After the mixture was heated for 10 min at 94°C, PCR was performed for 45 cycles at 94°C, at the appropriate annealing temperature and at 72°C for 1 min each, followed by 72°C for 10 min. After denaturation of the PCR products at 94°C for 2 min, samples were subjected to electrophoresis using Performance Optimised Polymer 4 in a 310 Genetic Analyzer (Applied Biosystems, Foster, CA, USA). Loss of heterozygosity analysis was performed by Gene Scan version 2.1. Loss of heterozygosity was quantitatively assessed by calculating the LOH index, which was defined as the allele ratio in the normal tissue divided by the allele ratio in the tumour tissue. The allele ratio was calculated as the peak height of the smaller allele divided by the peak height of the larger allele. If the LOH index was less than 0.5 or more than 2.0, we defined the case as LOH. The LOH frequency of each locus was represented by the ratio of the number of cases with LOH to the total number of informative cases.

### Immunohistochemical analysis of p53, Ki-67, apoptosis and microvessel density

Immunohistochemical analysis was conducted by avidin–biotin complex method using 2.5-*μ*m-thick sections from the paraffin-embedded specimens. For the Ki-67 detection, anti-human MIB1 monoclonal antibody (MoAb) (Immunotech S.A., Marseille, France) was used. After blocking the endogenous peroxidase activity, the sections were heated for 20 min in a microwave oven at 500 W to invigorate the antigens of the specimens, as reported by Shi *et al* (1991) with slight modification. DO-7 and CD34 MoAb's were used to immunostain p53 and microvessels in ovarian carcinomas using the standard immunoperoxidase procedure (Vectastain Elite ABC kit, Vector, Burlingame, CA, USA). Immunostaining and evaluation of p53 and microvessel density were performed as described previously (Nakayama *et al*, 2002, 2003a). Apoptotic cells were identified by terminal deoxynucleotidyl transferase-mediated dUTP-biotin nick end-labelling (TUNEL) methodology, using the Apop Tag *in situ* detection kit (Oncor, Gaithersburg, MD, USA). Terminal deoxynucleotidyl transferase-mediated dUTP-biotin nick end-labelling methods were performed as described previously (Hata *et al*, 2002). To obtain the AI (the percentage of immunostained apoptotic tumour cells) and Ki-67 labelling index (Ki-67 LI) (the percentage of immunostained tumour cells), 1000 tumour cells were examined by two observers (KN and YT).

### Statistical analysis

Progression-free survival time was measured in months from the month of surgery to the reported recurrence. Survival curves were determined using the Kaplan–Meier method, and differences in survival between subgroups were compared with the log-rank test. *P*-values less than 0.05 were considered significant. All reported *P*-values are two-sided.

## RESULTS

### LOH study

The frequency of LOH for each marker in informative tumours ranged from 0 to 27.3%, with an average of 8.6±4.3% (mean±s.d.). When 13% (mean+1 s.d.) or more of the tumours from informative cases showed LOH, we classified the LOH frequency of the locus as significantly high. Among 116 loci, we observed 22 loci with a significantly high LOH frequency (Table 2

**Table 2:** Microsatellite marker loci demonstrating significant frequency of LOH

**Locus**	**Localization**	**LOH (%)**^a^
D2S427	2q36	6/26 (23.1)
D3S2403	3p25	4/18 (22.2)
D3S1764	3q21	3/22 (13.6)
D5S1473	5q14	5/24 (20.1)
*D5S644*^b^	5q14–21	4/24 (16.6)
D5S1456	5q35	4/30 (13.3)
*D6S300*	6q14	3/23 (13.0)
D6S1003	6q24	3/11 (27.2)
*D6S473*	6q25	3/20 (15.0)
D6S1227	6q25	3/18 (16.7)
D6S503	6q27	3/11 (27.3)
D7S1805	7q35	6/31 (19.4)
D8S1119	8q21.2	4/27 (14.8)
D8S373	8q24.3	4/30 (13.3)
D9S922	9q21.32	4/25 (16.0)
D10S1435	10p15	3/21 (14.3)
*TEL*	12p12.3	4/23 (17.4)
*NFI*	17q11.2	5/26 (19.2)
***D19S433***^c^	19q12	4/25 (16.0)
DXS6807	Xp22.3	3/13 (23.6)
DXS6797	Xq23	4/26 (15.4)
***AR***	Xq11–12	3/17 (17.6)

a Cases with LOH/informative cases.

b Loci shown in italic reveal that markers reported to be associated with ovarian carcinoma.

c Loci shown in bold reveal that tumour with LOH at these loci correlate with adverse outcome.

LOH=loss of heterozygosity.

).

The most frequent LOH in mucinous LMPs was found at locus D6S503 (6q27; 27.3%).

### Clinicopathological factors and immunohistochemical study

The following variables were examined for association with disease progression by Kaplan–Meier methods: age (44> *vs* 44⩽); stage (Ia *vs* Ib⩽); CA125 (35> *vs* 35⩽); and tumour size (15 cm> *vs* 15⩽) (Table 3

**Table 3:** Univariate analysis association of study variables with progression

**Variables**	***n***	**Progression rate**	***P*-value**
*Age (years)*	41		
<44	20	0.15	0.3277
⩾44	21	0.19	

*Stage*	41		
Ia	30	0.03	<0.0001
⩾Ib	11	0.45	

*CA125*	41		
<35	21	0.19	0.1527
⩾35	20	0.15	

*T (tumour size)*	41		
<15	23	0.13	0.7686
⩾15	18	0.22	

*MVD*	21		
High	21	0.33	0.9326
Low	20	0.15	

*Ki-67 LI*	41		
High	21	0.19	0.042
Low	20	0.13	

*Apoptotic LI*	41		
High	21	0.14	0.3489
Low	20	0.2	

*p53 overexpression*	41		
Positive	2	0.5	0.0559
Negative	39	0.15	
			
*19q12 LOH*	25					
LOH	4	0.75	0.0023			
Retention	21	0.05				
			
*Xq11–12 LOH*	17					
LOH	3	0.67	0.0122			
Retention	14	0.05				
			
*19q12 and/or Xq11–12 LOH*	33					
LOH	5	0.8	<0.0001			
Retention	28	0.04				

LOH=loss of heterozygosity.

). The mean AI of the 41 mucinous LMPs was 1.6% (range, 0–5.6%; median, 1.3%). The proliferative activity of tumour cells is described by the Ki-67 LI in each case. The mean Ki-67 LI of the 41 mucinous LMPs was 28.0% (range, 0.6–77.4%; median, 26.0%). The mean MVD of the 41 mucinous LMPs was 8.3% (range, 3.0–41.0%; median, 6.5%). Overexpression of p53 by immunohistochemistry was observed in 4.2% (two of 41) of analysed tumours. Concerning AI, Ki-67 LI, and MVD, we used the median value (1.3, 26.0, and 6.5) as the optimal cutoff point to divide the patients into two groups (Table 3). As the cases with detectable p53 overexpression were rare, we used positive or negative expression as the optimal cutoff point to divide the patients into two groups.

### Progression risk

**Figure 3:**
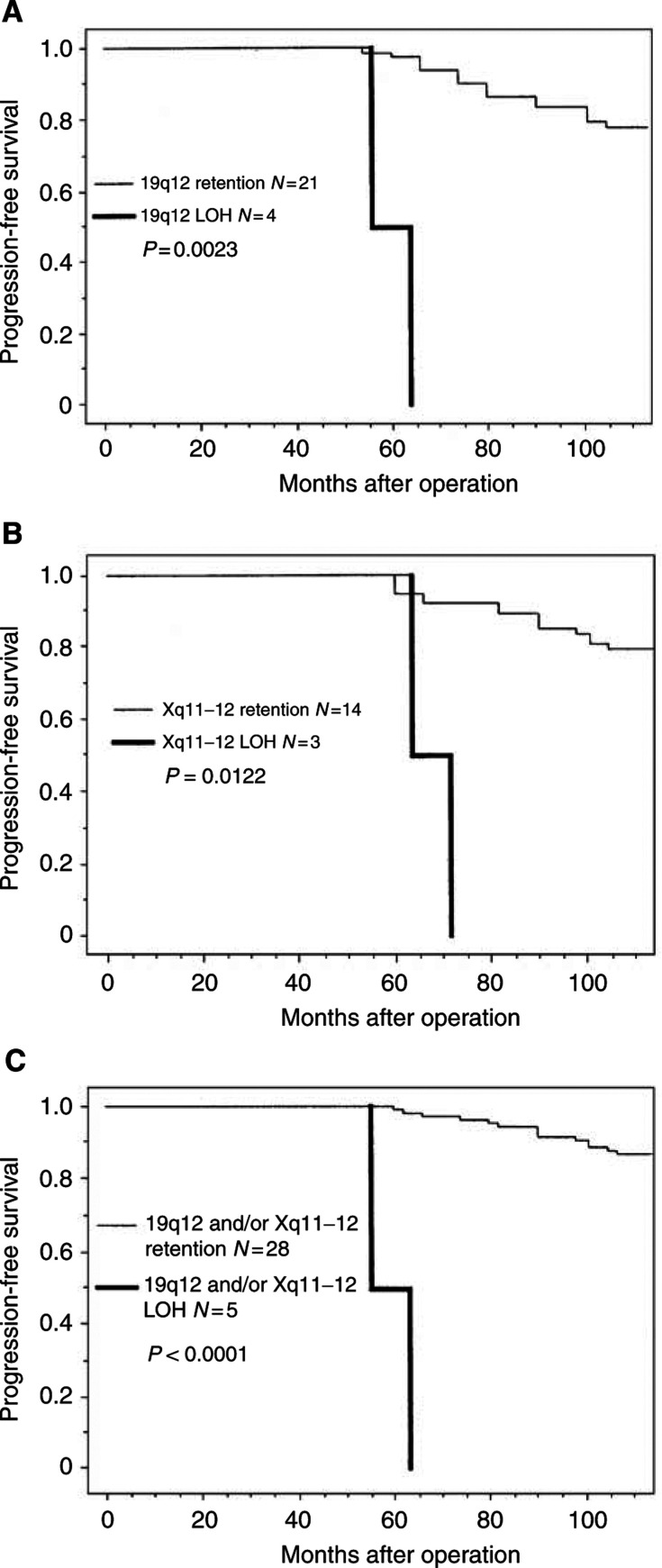
Kaplan–Meier curves for progression-free survival time of patients with tumours that retained both alleles (retention) or that had lost one allele (LOH) at a marker locus. LOH at 19q12 (**A**), at Xq11–12 (**B**), and at 19q12 and/or Xq11–12 (**C**) was significantly associated with progression-free survival time.

Of the 116 markers examined, 19q12(D19S433) and Xq11–12 (AR) showed significant correlation between postoperative progression-free survival time and LOH status (Table 3). Kaplan–Meier analysis of progression-free survival time revealed an increased risk of postoperative progression in patients with tumours exhibiting LOH at 19q12 and Xq11–12 compared to patients with tumours that retained both alleles at these loci. As shown in Table 1, we analysed the postoperative progression rate with regard to LOH status at each of the loci, and carried out a log-rank test to evaluate the statistical significance by univariate analysis. None of the other 114 markers tested were correlated with prognosis. Interestingly, these two loci were classified into the significantly high LOH group (16.0%, four of 25, 17.6%, three of 17, respectively). Of the 41 patients with tumours informative at 19q12, 75% of those with LOH progressed within 5 years of surgery, compared to a 5% progression rate among patients with tumours that retained both alleles (*P*=0.0023, Figure 3A

). A correlation between LOH and progression was also observed at Xq11–12, with a 5-year progression rate of 67% in patients with LOH and 5% in patients without LOH (*P*=0.0122, Figure 3B). Likewise, a combination of 19q12 and/or Xq11–12 revealed a 5-year progression rate of 80% in patients with LOH and 4% in patients without LOH (*P*<0.0001, Figure 3C). All comparisons were significant (Table 3). In contrast, patients with a high Ki-67 LI had a significantly poorer progression-free survival time than those with lower levels (*P*=0.042, Table 3). Other clinicopathological factors and immunohistochemical analysis had no correlation with progression-free survival time in this series of patients (Table 3). There was no significant difference in progression-free survival time between intestinal and endocervical type (data not shown). To determine whether these factors were prognostic marker(s) independent of FIGO stage, an established prognostic marker, we conducted a progression-free survival time analysis using the Cox's proportional-hazards model (Table 4

**Table 4:** Multivariate analysis of progression-free survival in LMP patients

	**Univariate**	**Multivariate**		
**Variables**	***P*-value**	**Hazard ratio**	**95% CI**	***P*-value**
*Stage*				
Ia (*n*=30) *vs* ⩾Ib (*n*=11)	<0.0001	4.5	1.0–20.4	0.0484

*Ki-67 LI*				
High (*n*=21) *vs* low (*n*=20)	0.042	1.5	0.5–4.2	0.4351

*19q12 and/or Xq11–12 LOH*				
LOH (*n*=5) *vs* retention (*n*=28)	<0.0001	32.7	2.6–417.1	0.0073

LOH=loss of heterozygosity; LMP=low malignant potential.

). When the combination of LOH at 19q12 and/or Xq11–12 was assessed, patients with tumours who showed LOH at those positions were at greatest risk of progression (*P*=0.0073, Table 4).

## DISCUSSION

Mucious LMPs are often associated with a significantly better prognosis than mucinous ovarian carcinomas (Siriaunkgul *et al*, 1995; Pecorelli and Fallo, 1998; Lee and Scully, 2000). However, gynaecologic oncologists have long sought prognostic markers that could help to assess accurately the postoperative progression risk in mucinous LMP patients, because a subset of these tumours can progress and become lethal. For advanced stage mucinous LMPs, earlier detection of a recurrence may not always result in improved prognosis, due to the lack of efficacious chemotherapy (Tamakoshi *et al*, 1997). However, in patients with early-stage tumours who have been treated primarily with conservative surgery, earlier detection of recurrence may result in better prognosis. Although until recently routine postoperative decisions for mucinous LMP patients rely on the consideration of conventional prognostic factors such as clinical stage, recently, Winter *et al* (2002) reported that survival and recurrence rates were not significantly different between staged and unstaged patients who had surgery for LMP patients. Only about half of all patients found to have LMPs undergo complete surgical staging (Link *et al*, 1996). Removal of the appendix is often included in the staging procedure for ovarian carcinoma, including LMP lesions. The occurrence of synchronous mucinous tumours of the ovary and appendix has been reported (Young *et al*, 1991; Seidman *et al*, 1993; Prayson *et al*, 1994). Recent studies of cytokeratin immunostaining patterns, clonality studies with LOH, and molecular studies of K*-ras* mutations have supported the conclusion that *Pseudomyxoma peritonei* usually takes origin from the appendix and not the ovary (Guerrieri *et al*, 1995, 1997; Cuatrecasas *et al*, 1996; Szych *et al*, 1999). In the current study, only one case had a recurrence as *Pseudomyxoma peritonei*, but we diagnosed it as of ovarian origin because the tumour was restricted to the left ovary and the appendix that was dissected during the surgical operation was intact.

In the present study, we assessed immunohistochemical analysis and various clinicopathological factors that could predict the postoperative progression risk for mucinous LMP patients. However, age, histology, CA125 levels, tumour size, MVD, apoptotic LI, and p53 overexpression did not correlate with postoperative progression rate. Previous studies have suggested that DNA ploidy is prognostically important in mucinous LMP patients (Kaern *et al*, 1990; Padberg *et al*, 1991). Kaern *et al* (1990) and Padberg *et al* (1991) have correlated DNA aneuploidy with increased tumour progression and shortened patient survival time. In the present study, high Ki-67 LI was significantly associated with tumour progression. Proliferative activity assessed by Ki-67 LI was significantly correlated with DNA aneuploidy in ovarian neoplasm (Huettner *et al*, 1992). Therefore, Ki-67 LI may indirectly reflect DNA aneuploidy in this series of mucinous LMPs.

Recently, specific LOH patterns have been implicated as predictors of poor survival in various solid tumours (Choi *et al*, 2000; Hirano *et al*, 2001; Choi *et al*, 2002; Rosin *et al*, 2002; Nakayama *et al*, 2003b). In the current study, of the 116 markers examined only 19q12 and Xq11–12 showed significant correlation between postoperative progression-free survival time and LOH status (Table 3).

We found that patients whose tumours had shown LOH at 19q12 had a significantly shorter progression-free survival time than those with retention of both alleles at 19q12. Although deletions of chromosome 19q12 have been reported in head and neck carcinoma, gliolbastoma, and astrocytoma, the association of LOH at that region with poor survival has not been reported previously (Nunn *et al*, 1999; Hu *et al*, 2002; Beder *et al*, 2003). We speculate that a candidate tumour suppressor gene in the 19q12 region is associated not only with tumour progression but also with an aggressive clinical phenotype.

At the genetic level, mucinous neoplasms show a characteristic profile that is different from those of serous neoplasms. K-*ras* mutation is highly characteristic of ovarian mucinous lesions, and may indeed be the defining mutation that confers upon the lesion a mucinous phenotype (Mandai *et al*, 1998). Schwartz *et al* (2002) showed that mucinous ovarian carcinomas can be readily distinguished from serous ovarian carcinomas based on their gene expression profiles using oligonucleotide microarrays, regardless of tumour stage and grade (). Furthermore, the LOH profile of mucinous LMPs is similar to that of mucinous ovarian carcinomas, and in tumours showing LMP and invasive carcinoma components, common LOH events have been demonstrated between the LMP and carcinoma areas (Nakayama *et al*, 2001). These suggest that mucinous LMPs in some cases progress to invasive carcinomas. It has been suggested that deletions that include the AR locus at Xq11.2–12 are involved in the development of both LMP and ovarian carcinoma, including mucinous subtype (Edelson *et al*, 1998). Thus, mucinous LMP tumours with LOH at that region are precursors of mucinous ovarian carcinoma and result in a poor clinical outcome. Since these two loci discussed above were classified into the significantly high LOH group (16.0%, four of 25, 17.6%, three of 17, respectively, Table 2) in the present allelotype analysis, inactivation of a candidate tumour suppressor gene around these regions could be crucial for the tumourigenesis of mucinous LMP.

This study had several limitations. One limitation is that the number of patients studied was relatively small because of the logistics of long-term patient follow-up. Another limitation is that *Pseudomyxoma peritonei* related to mucinous LMP that is more likely to be associated with advanced stage, recurrence, and poor prognosis (Nakashima *et al*, 1990; Casey *et al*, 1993) was included in only one case in this study. A third limitation is that the multifocality of metastatic disease was included only one case in this study. The fourth limitation is that LMPs with microinvasion was not included in this series of tumours. Larger studies with more cases, including *Pseudomyxoma peritonei* and LMPs with microinvasion, are needed to clarify the significance of LOH at 19q12 and Xq11–12.

In summary, the present study described for the first time the use of microsatellite analysis to predict the poor outcome for mucinous LMP patients. The results of our study suggest that the identification of LOH at 19q12 and/or Xq11–12 in former mucinous LMP sites should alert the clinician to the presence of a potentially aggressive lesion in the coelomic epithelium, even if the distinction between second primary tumours or recurrence could not be determined.
